# Evaluation of Bcl-2 as a marker for chronic kidney disease prediction in cats

**DOI:** 10.3389/fvets.2022.1043848

**Published:** 2023-01-09

**Authors:** Pattiya Pila, Phongsakorn Chuammitri, Prapas Patchanee, Kidsadagon Pringproa, Kakanang Piyarungsri

**Affiliations:** ^1^Department of Companion Animal and Wildlife Clinic, Faculty of Veterinary Medicine, Chiang Mai University, Chiang Mai, Thailand; ^2^Research Center of Producing and Development of Products and Innovations for Animal Health and Production, Chiang Mai University, Chiang Mai, Thailand; ^3^Department of Veterinary Bioscience and Veterinary Public Health, Faculty of Veterinary Medicine, Chiang Mai University, Chiang Mai, Thailand; ^4^Department of Food Animal Clinic, Faculty of Veterinary Medicine, Chiang Mai University, Chiang Mai, Thailand

**Keywords:** Bcl-2, biomarker, cat, chronic kidney disease, prediction

## Abstract

Chronic kidney disease (CKD) is a frequent condition in elderly cats. Bcl-2 is linked to kidney disease through the processes of apoptosis and fibrosis. The purpose of this study is to examine Bcl-2 levels in CKD and clinically healthy age-matched cats in order to evaluate the relationship between Bcl-2 levels, signalment, and blood parameters in cats with CKD. The circulating levels of Bcl-2 were determined using an immunoassay in twenty-four CKD cats and eleven clinically healthy age-matched cats by the utilization of the general linear model (GLM), Pearson correlation, principal component analysis (PCA), ROC curves, the Cox hazard model, and Kaplan–Meier survival analysis. These were all conducted in order to explore Bcl-2 levels and their connection with other variables. The Bcl-2 immunohistochemical intensity was graded in each glomerulus and tubulointerstitium. McNemar's test was performed in order to compare the expression of Bcl-2 in the two renal tissue sites. The circulating Bcl-2 of CKD cats was significantly lower than those of clinically healthy age-matched cats (*P* = 0.034). The presence of circulating Bcl-2 (*P* < 0.01) and the severity of CKD (*P* = 0.02) were both linked with the survival time of cats with CKD. The area under the curve (AUC) of Bcl-2 for detection of CKD was 0.723. In cats, decreased circulating Bcl-2 was associated with increased blood BUN, creatinine levels, and CKD severity. Bcl-2 protein expression was reduced in the renal tissues of CKD cats as the disease progressed, resulting in a decrease in their survival time. This study demonstrated that Bcl-2 may be effective in diagnosing feline CKD.

## Introduction

Chronic kidney disease (CKD) is a common disease in older cats. CKD can affect cats of various ages and breeds (1). The prevalence of feline CKD was 1.9% in the United States (2), 20% in Australia (3), as well as 0.63 and 2.37% in Bangkok and Chiang Mai, Thailand, respectively (4, 5). Feline CKD operates in four stages based on serum creatinine and symmetric dimethylarginine (SDMA) (6). Additionally, sub-staging is possible due to proteinuria and hypertension.

The glomerular filtration rate (GFR) is used to assess renal function. Inulin clearance is the gold standard for the purposes of measuring GFR. However, this method is impractical and time-consuming. Although serum creatinine remains a criterion for determining GFR, it is normal until GRF is reduced by nearly 75%. Moreover, it can be influenced by non-renal factors, such as muscle mass.

The Bcl-2 family of proteins plays a critical role in regulating the apoptosis process. The Bcl-2 family proteins are regulated by the intrinsic pathway of apoptosis, which can be activated by DNA damage, oxidative stress, radiation, oncogene activation, and nutrient deprivation. There are three classes of Bcl-2 family proteins—anti-apoptosis, pro-apoptosis, and BH3-only proteins—that bind and regulate the anti-apoptotic in order to promote apoptosis (7). Bcl-2 is an anti-apoptotic gene that has an inverse correlation with Bax, as shown in the previous study of unilateral ureteral obstruction (UUO) subjects, whereby it was shown that Bcl-2 levels will decrease during UUO (8, 9). Bcl-2 levels of patients with end-stage renal disease, who were maintained on hemodialysis, underwent decreased expression when compared to the CKD group (10). A lack of Bcl-2 will increase the expression of Bax, which promotes the release of cytochrome C and mitochondrial outer membrane fragmentation. Further, the expression of Bax will also lead to caspase-3 activation and cell death (7, 8). In another study of humans, it was found that a deposition of myofibroblasts at the interstitial area after cell death can induce irreversible kidney damage. Moreover, kidney fibrosis is known to also result in chronic kidney disease (11).

However, there have been a few studies that have investigated Bcl-2 levels in cats with CKD. Thus, this study aimed to compare the Bcl-2 levels between CKD and clinically healthy age-matched cats. This was performed in order to determine the correlation among the Bcl-2 levels, signalment, and blood parameters of cats with CKD, as well as to determine the Bcl-2 level in the kidney tissues of CKD cats—in both quantitative and qualitative aspects *via* immunohistochemistry techniques.

## Materials and methods

### Animals and sample collection

Twenty-four CKD and 11 clinically normal age-matched cats were included. The CKD cats were presented at the Small Animal Hospital, Faculty of Veterinary Medicine, Chiang Mai University. There were no restrictions on gender or breed for these CKD cats. However, the age must be ≥5 years old with serum creatinine levels >1.6 mg/dl; a urine specific gravity at < 1.035; and blood urea nitrogen levels >35 mg/dl, or with abnormal kidney structure that is revealed by ultrasound, such as in the small kidney, polycystic kidney, and hydronephrosis. Moreover, CKD cats must have azotemia or abnormal kidney structure for more than 3 months before being included in the study. They were also excluded if they had hyperthyroidism, other thyroid-related problems, or if they were fed a phosphorous-restricted diet. In addition, 11 clinically normal age-matched cats were examined and none were found to have any signs of CKD. Furthermore, individuals must pass a routine physical examination and additional blood chemistry tests in order to be eligible. All the clinically normal age-matched cats had serum SDMA levels < 14 μg/dL, which was within the reference interval. In addition, the clinically normal age-matched cats were randomly selected without regard to gender or breed. The exclusion criteria of CKD and healthy cats were set as those cats that possessed other systemic diseases and/or retrovirus infections. The following ethics committee approved all cats for animal experimentation: the Faculty of Veterinary Medicine, Chiang Mai University No. S25/2563.

**Figure 1:**
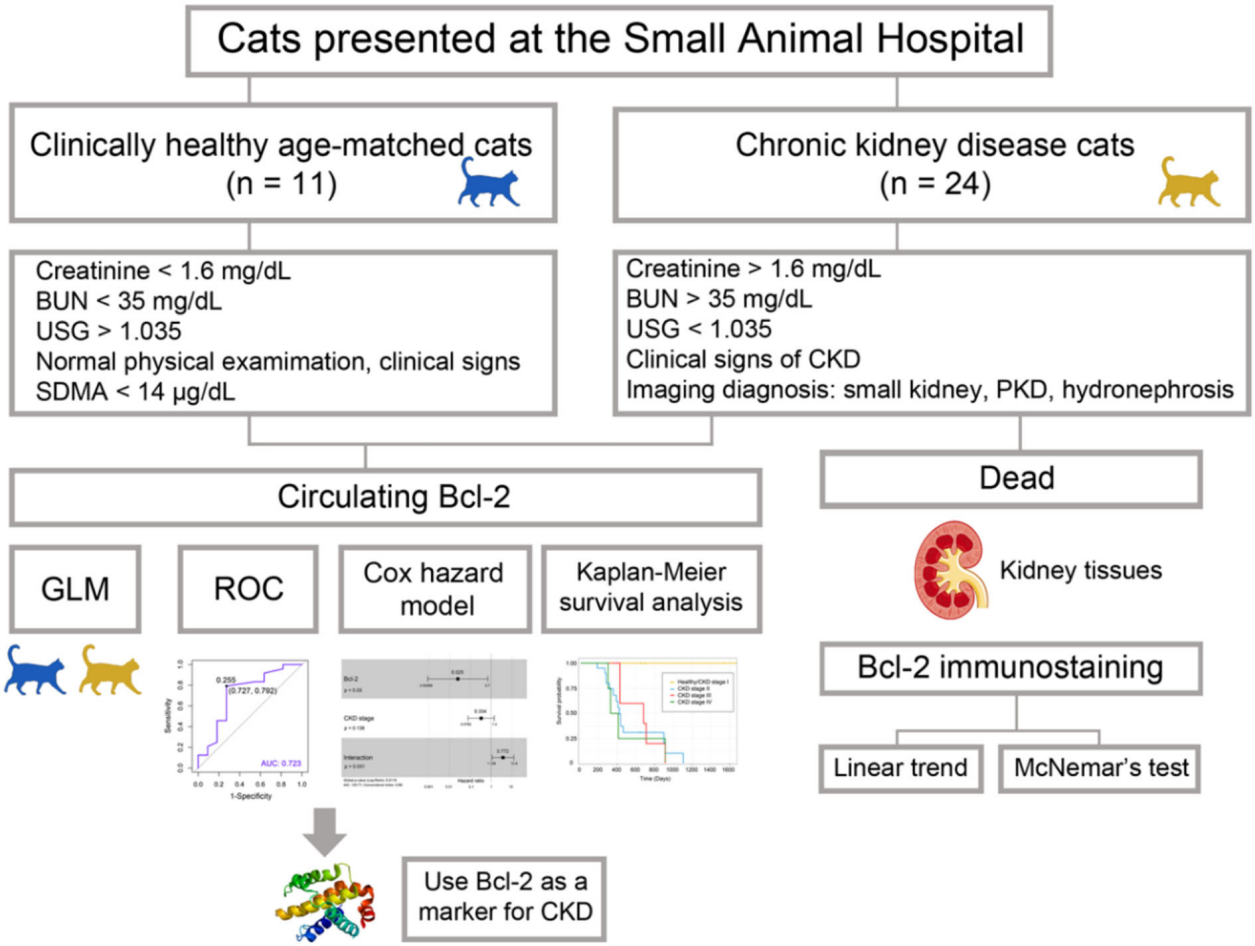
The workflow of the experiment. The goal of Bcl-2 studies is to determine the efficacy of Bcl-2 as a biomarker in feline chronic renal disease.

Blood and urine samples were collected from both groups. Blood samples were collected from the cephalic or saphenous vein. They were stored in EDTA tubes (0.5 ml) and heparin tubes (1 ml) for the purposes of analyzing complete blood count and blood chemistry. One ml of each blood sample was collected in VACUETTE^®^ serum tubes (Greiner Bio-One, Kremsmünster, Austria) in order to obtain serum for the purposes of determining the circulating Bcl-2. Serum samples were stored at −20°C for further analysis. The cystocentesis procedure was used to obtain urine samples for urinalysis analysis. Seventeen kidney tissues were obtained from cats that died during the study from CKD at the Small Animal Hospital, Faculty of Veterinary Medicine, Chiang Mai University. These kidney tissues were analyzed for the purposes of evaluating the Bcl-2 immunostaining intensity. The conceptual framework of our investigation is shown schematically in Figure 1.

### Dot blot analysis

Cat sera were diluted with PBS (1:1) and incubated at 95°C for 5 min. The PVDF membrane was soaked using methanol and placed on soaked Whatman filter paper. Three μl of diluted serum was dropped onto the PVDF (Bio-Rad, Hercules, CA, USA) and dried. Then, it was blocked with a blocking buffer for 30 min at room temperature. After that, the PVDF was incubated with a primary mouse monoclonal anti-Bcl-2 antibody (BioLegend, San Diego, CA, USA) (1:1,000 dilution) within a blocking buffer at room temperature for 2 h. It was then incubated with HRP secondary goat anti-mouse IgG (BioLegend) (1:2,000 dilution) in a blocking buffer for 45 min at room temperature. Finally, the membrane was stained with a DAB substrate and dried at room temperature. Another PVDF membrane was prepared for the purposes of determining the total protein in each serum. This PVDF has been stained with Ponceau S (Sigma-Aldrich, St. Louis, MO, USA) staining for 10 min at room temperature. The Image Studio Lite^TM^ program (LI-COR, Lincoln, NE, USA) was used for the purposes of quantifying the densitometry of proteins, which were in the ratio of Bcl-2/Ponceau S (12, 13). Each typical dot blot was converted to grayscale before protein densitometry was quantified.

### Immunohistochemistry

Kidney tissues were obtained from the autopsy unit in the Faculty of Veterinary Medicine, Chiang Mai University. Each slide was deparaffinated *via* xylene for 10 min. Then, it was rehydrated by absolute ethanol, 90% ethanol, 70% ethanol, and 50% ethanol for 5 min/each step. Next, each slide was warmed with a citrate buffer for the purposes of antigen retrieval. The slices were arranged in a container with citric buffer at a pH of 6.0 and incubated in the microwave, 700 w for 20 min. After that, the slides were blocked with endogenous peroxidase in 3% hydrogen peroxide for 5 min and washed with PBST three times for 5 min each. The 200 μl of 2.5% bovine serum albumin was added onto each slide and incubated for 5 min. The primary antibody was applied *via* dropping 200 μl of a primary antibody that was diluted with 1% PBS to the sections of the slides and then incubating at 37 °C for 2 h. The slide was incubated with a primary mouse monoclonal anti-Bcl-2 antibody (BioLegend) (1:200 dilution) in a blocking buffer at 37 °C for 2 h and then all slides were washed with PBS, three times for 5 min. The 200 μl of normal goat serum (1:5) was added at room temperature for 30 min and washed with PBS, three times for 5 min. Then, 200 μl of HRP secondary goat anti-mouse IgG (BioLegend) (1:200 dilution) in a blocking buffer was added for 45 min at room temperature and then washed with PBS, three times for 5 min. DAB substrate was used for the purposes of staining for 5 min, as well as terminating the reaction by rinsing with running tap water. The slides were stained with hematoxylin for 10 s, lithium for 10 s, and then washed with distilled water. Finally, the process of mounting and covering the slides was performed. Negative control was conducted *via* replacing the primary antibody with the normal mouse serum (1:200). The immunostaining was visualized and scanned with a Pannoramic MIDI Slide Scanner (3D HISTECH, Budapest, Hungary). The scoring basis was semi-quantitative and was scored from 0 to 2 (14), as follows: grade 0 is no staining; grade 0.5 is trace staining; grade 1 is weak staining; and grade 2 is strong staining. The following equation was utilized in order to determine the score:


$$\begin{array}{l} \text{Intensityscore}=\left[\left(0.5\times {\text{N}}_{0.5}\right)+\left(1\times {\text{N}}_{1}\right)+\left(2\times {\text{N}}_{2}\right)\right]/\text{N} \end{array}$$

### Statistical analyses

The circulating Bcl-2 levels between CKD and clinically normal age-matched cats were compared using the general linear model (GLM). The correlation among the circulating Bcl-2, signalment, and blood parameters was determined using Pearson's correlation method. The association between the survival time of the CKD cats and the potential variables was investigated using the Cox hazard model and Kaplan–Meier survival analysis. The area under the curve of Bcl-2 for the purposes of detecting CKD was achieved through using the receiver operating characteristic. In regard to statistical testing and graphical presentations, the following R packages were used: cluster, corrplot, factoextra, ggfortify, ggplot2, pROC, RColorBrewer, survival, survminer, and viridis. In order to identify factors linked with the progression of CKD, we employed a Cox proportional hazard model. Moreover, a backward-stepwise Cox regression analysis was performed on all variables with a *p* < 0.20. The linear trend estimates the intercepts and slopes of mean trends for the CKD stage and the Bcl-2 IHC staining. Furthermore, McNemar's test was used for comparing the Bcl-2 expression of the two locations of the renal tissues; the result with *P* < 0.05 was considered significant. The GraphPad Prism 7 (GraphPad Software, San Diego, CA, USA) or R packages (The R Foundation) were used to visualize the data acquired from statistical analysis.

## Results

**Figure 2:**
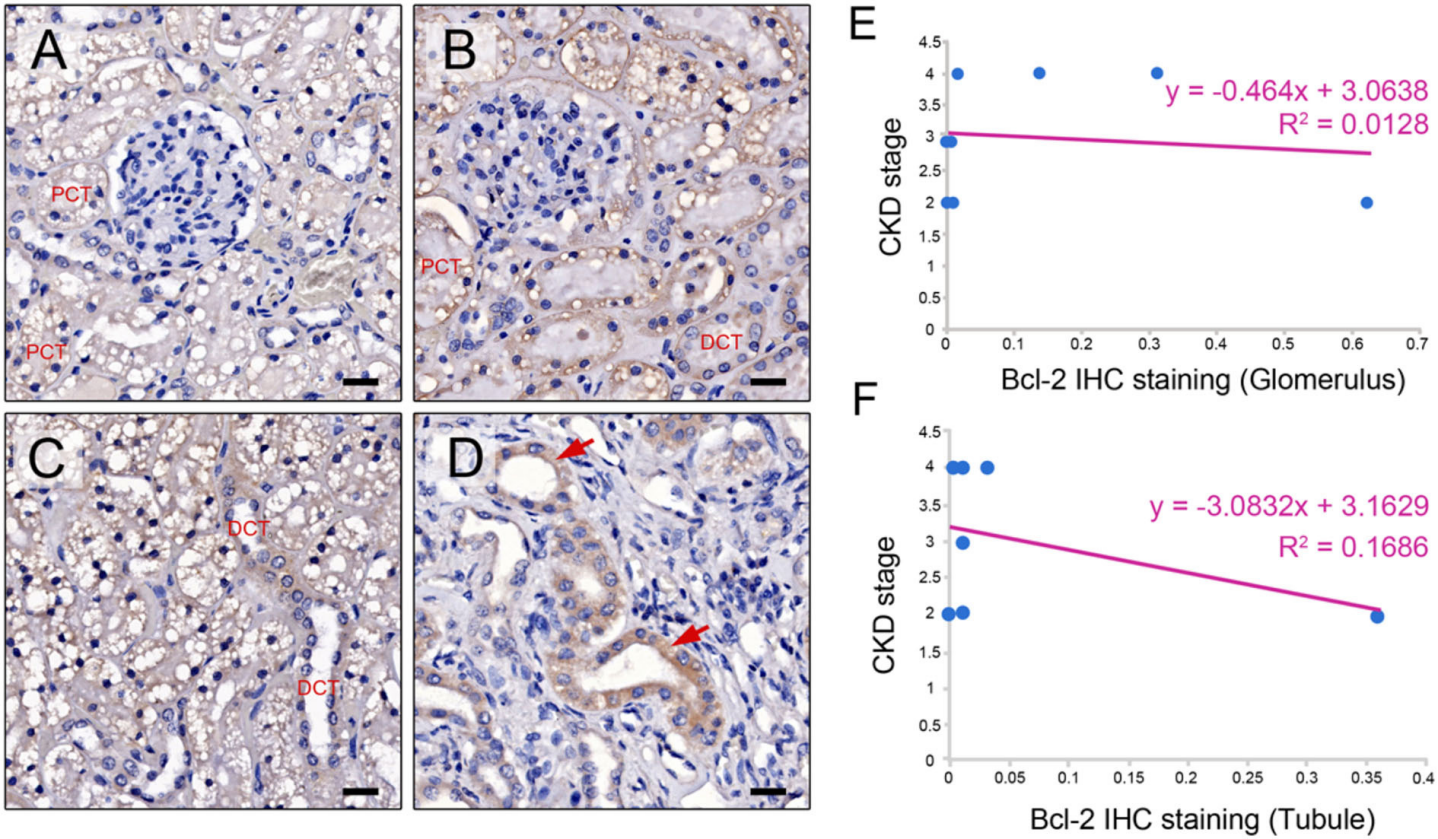
Bcl-2 protein expression. Representative IHC images illustrating the range of staining intensity for Bcl-2 in the Bowman's capsule, the proximal convoluted tubule (PCT), distal convoluted tubule (DCT) **(A, B)**, the distal convoluted tubule (DCT) **(C)**, and the collecting duct in the cats with CKD (red arrows) **(D)**. Original magnification 400 × . Scale bar = 20 μm. CKD stage and Bcl-2 staining are correlated at the glomerulus and **(E)** and tubule **(F)**.

The Bcl-2 immunostaining was detected within the CKD cat glomerular and tubular epithelium cells of the renal tissues (*n* = 17) (Figures 2A–D). When examined at the glomerular sites, Bcl-2 protein was found in the Bowman's capsule, the proximal convoluted tubule (PCT), and the distal convoluted tubule (DCT), as shown in Figures 2A, B. The Bcl-2 immunostaining was also identified in the distal convoluted tubule and the collecting duct at renal tubules (Figures 2C, D). There was no difference in the mean intensity of Bcl-2 staining at the glomerular (0.2 ± 0.27) and tubulointerstitium (0.19 ± 0.32).

The CKD stage and Bcl-2 staining had a negative connection. Furthermore, in the advanced stage of CKD, both glomerular and tubular Bcl-2 expression was reduced (Figures 2E, F). McNemar's test revealed that the renal tissues of the CKD cats contained Bcl-2 protein staining in the same proportion at the glomerular and tubular sites. As a result, Bcl-2 labeling at both sites did not affect disease progression. Furthermore, a negative control was demonstrated by substituting the primary antibody with normal mouse serum (Supplementary Figure 1).

**Figure 3:**
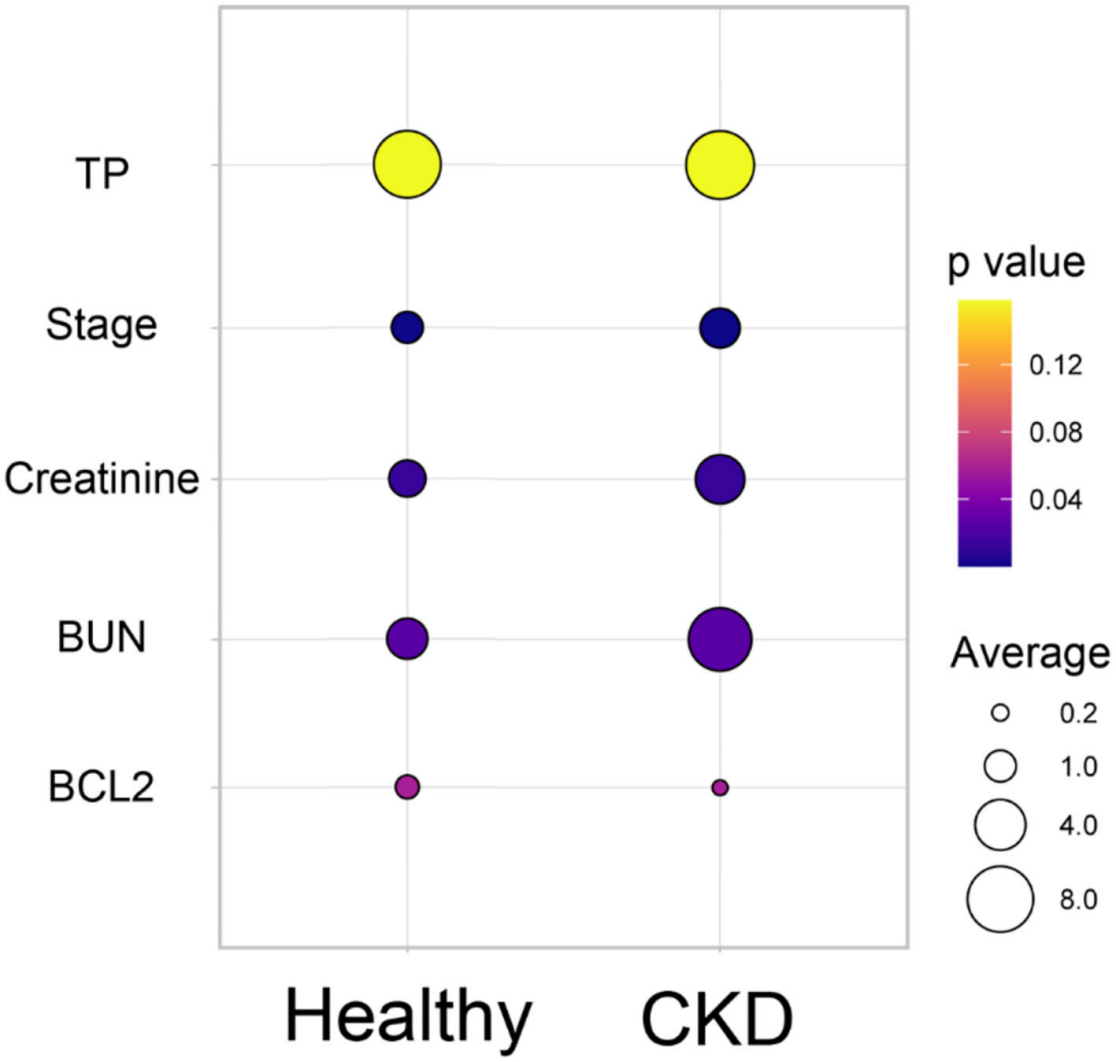
Bubble plot of the comparison of the average blood parameters between the clinically healthy age-matched and CKD cats. The average of each blood parameter is shown in different circle sizes. The *p*-value of the different averages of each group is shown in the different colors.

**Figure 4:**
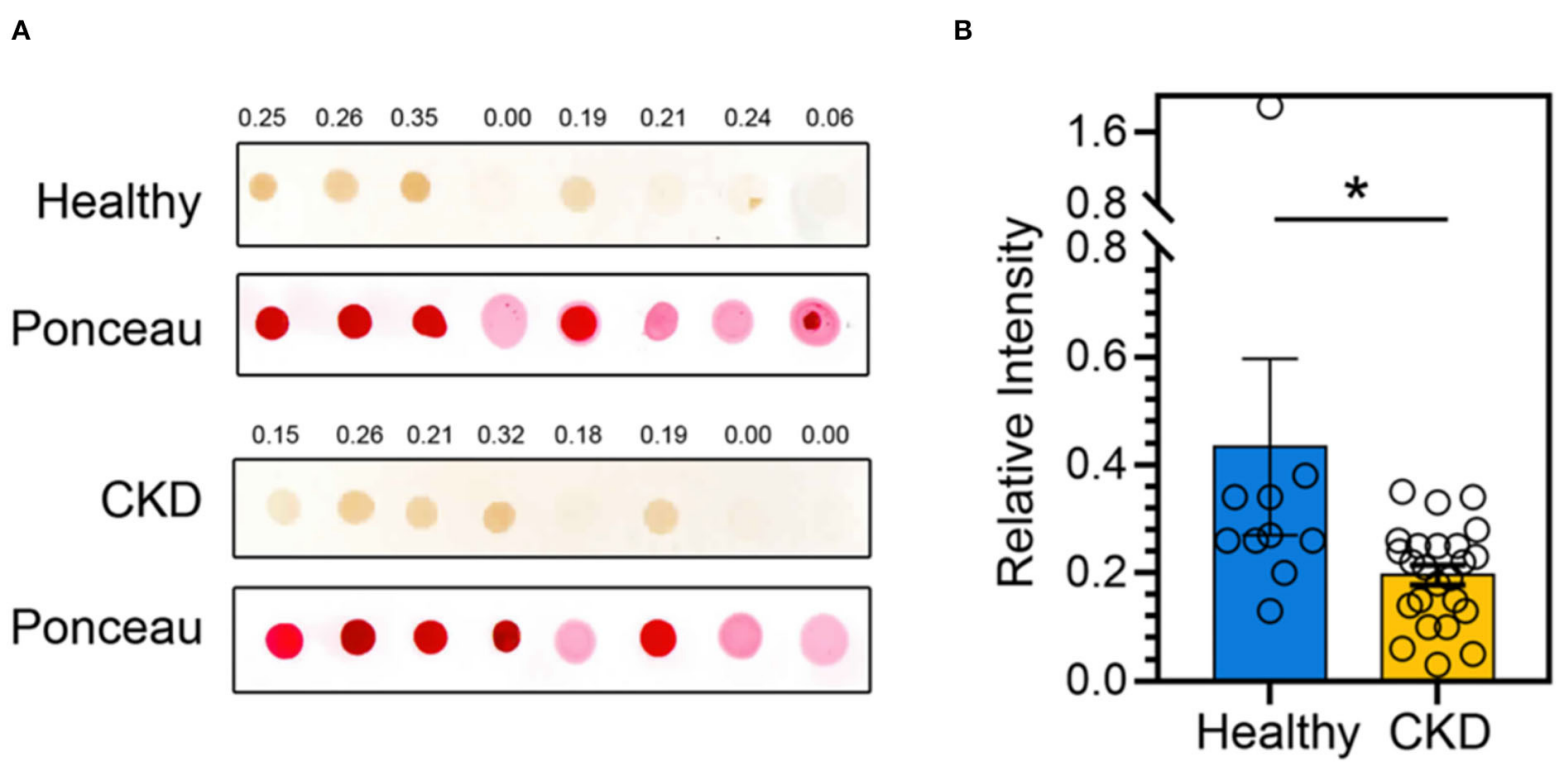
**(A)** A representative dot blot of the Bcl-2 abundance of clinically normal age-matched (upper panel) and CKD cats (lower panel). Ponceau S staining is used in order to control the amount of protein loaded onto membranes. Relative Bcl-2 intensities are indicated at the top of each panel. **(B)** The bar graph depicts the mean and the standard error of the mean (SEM) of relative Bcl-2 dot intensity of clinically normal age-matched (*n* = 11) and CKD cats (*n* = 24). *P < 0.05. Statistical significance was determined using a generalized linear model (GLM).

According to the comparison results of the average blood parameters between the clinically healthy age-matched and CKD cats, the CKD stage (*P* = 0.0001) was statistically different between the clinically normal age-matched and CKD cats, followed by creatinine (*P* = 0.02), BUN (*P* = 0.02), and Bcl-2 (*P* = 0.05) (Figure 3). The hematocrit of CKD cats was considerably lower than that of clinically normal age-matched cats (*P* < 0.01). Moreover, Bcl-2 levels in CKD cats were significantly lower than in clinically normal age-matched cats (*P* = 0.034); please refer to Table 1 and Figure 4A for more detail. In addition, Figure 4B depicts the Bcl-2 protein intensity in clinically normal age-matched and CKD cats.

**Table 1:** Mean ± standard deviations (SDs) of other parameters and relative Bcl-2 abundance between clinically normal age-matched and chronic kidney disease cats.

**Parameter**	**Unit**	**Clinically normal cats** **(*n =* 11)**	**CKD cats** **(*n =* 24)**	***P-*value**
Age	years	8.85 ± 2.26	9.76 ± 3.86	0.412
Body weight	kg	4.60 ± 1.03	4.46 ± 1.24	0.754
Systolic blood pressure	mmHg	146.67 ± 5.77	161.78 ± 27.57	0.155
BUN	mg/dL	21.21 ± 2.73	71.72 ± 70.05^**^	**0.002**
Creatinine	mg/dL	1.51 ± 0.09	3.68 ± 2.58^**^	**< 0.001**
Albumin	mg/dL	3.34 ± 0.22	3.23 ± 0.32	0.236
Phosphorous	mg/dL	3.38 ± 1.02	5.30 ± 4.01	0.067
Hematocrit	%	42.44 ± 3.91	32.41 ± 7.11^**^	**< 0.001**
Relative Bcl-2 abundance		0.43 ± 0.16	0.19 ± 0.02^*^	**0.034**

CKD, Chronic Kidney Disease; BUN, blood urea nitrogen.

^**^P < 0.01; ^*^P < 0.05.

The bold values refer to the parameters with a *P*-value less than 0.05.

**Figure 5:**
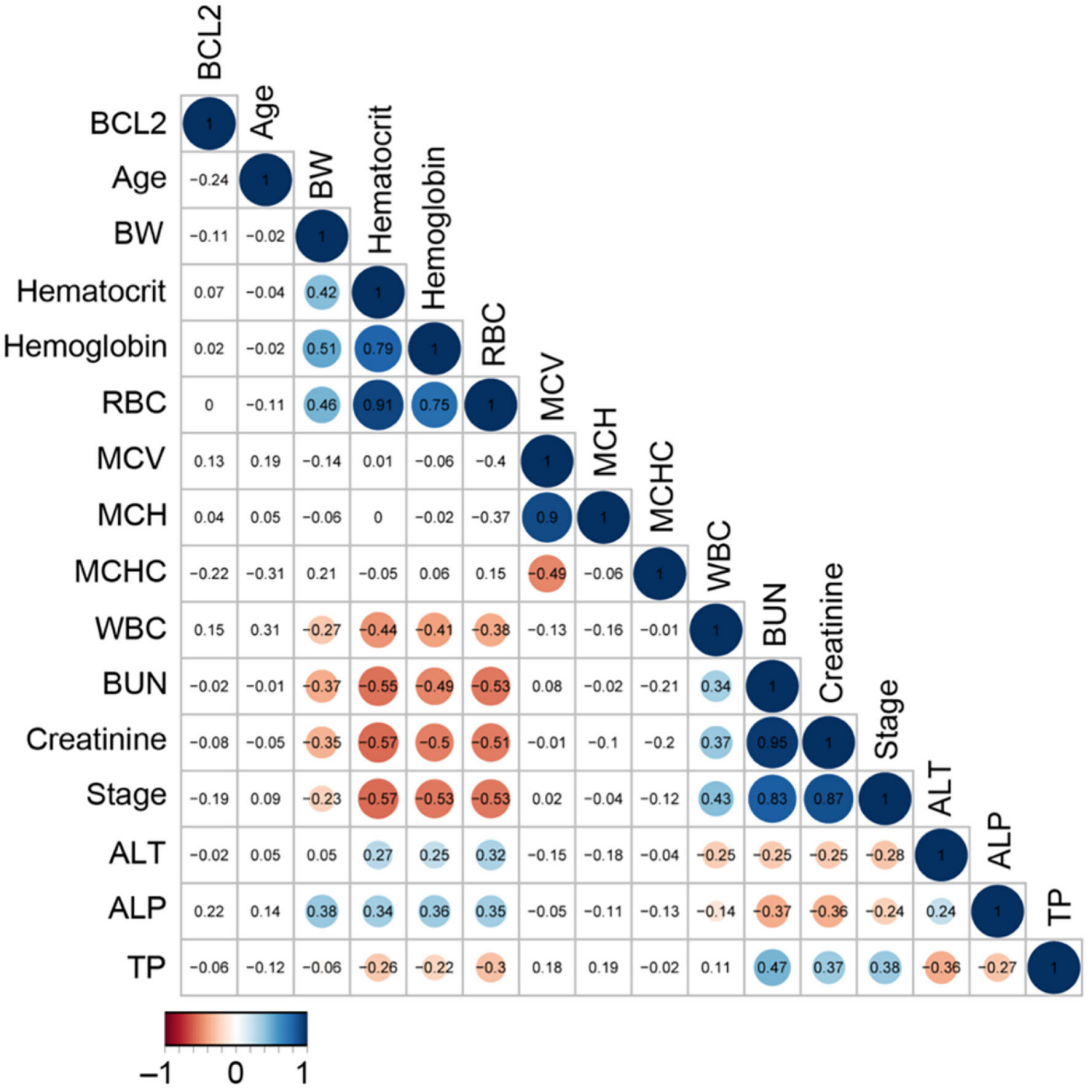
The correlation analysis of circulating Bcl-2 with other blood parameters. The correlation matrix's colored circles represent the correlation coefficients for each pair of Bcl-2 in the relevant cell-matrix.

The CKD stage was a positive correlation with BUN (r = 0.83), creatinine (r = 0.87), and leukocyte (r = 0.43), but a negative correlation with RBC (r = **–**0.53), hematocrit (r = **–**0.57), and hemoglobin (r = **–**0.53) (Figure 5). In order to analyze the principal component analysis (PCA), factors that interacted with CKD progression (such as circulating Bcl-2, blood urea nitrogen (BUN), creatinine, and kidney disease stage) were examined.

**Figure 6:**
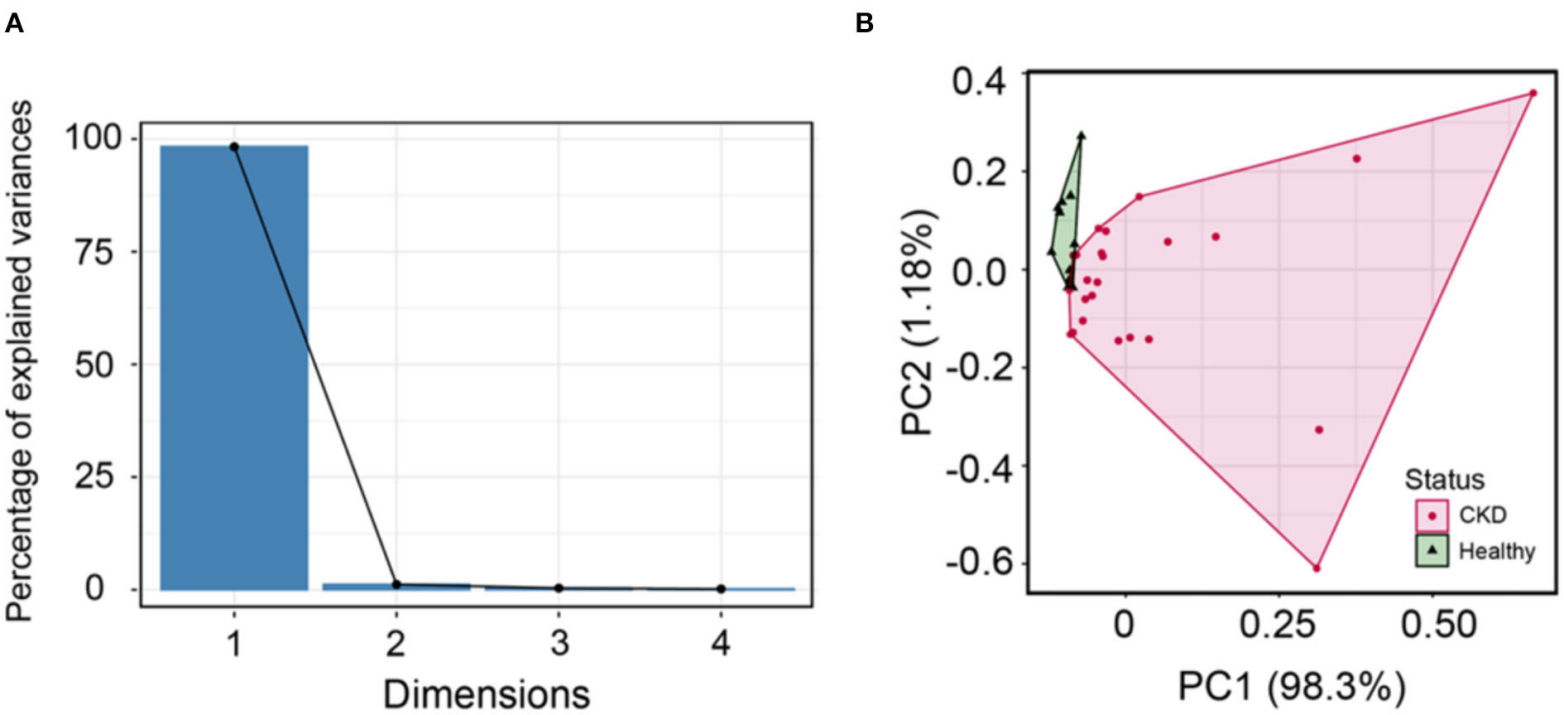
**(A)** The scree plot shows dimensions and percentage of variance, as explained by each dimension (PC). **(B)** Principal component analysis can distinguish between normal age-matched cats (green) and CKD (pink) detected when using cluster analysis.

A scree plot was employed in order to determine the number of dimensions to conduct in PCA. According to the scree plot, just two components (PC1 and PC2) were utilized to correlate variation in each component (Figure 6A). Further, PCA can be conducted with these two components. The circulating Bcl-2 can distinguish between CKD and clinically normal age-matched cats using PCA (Figure 6B). Although there were no correlations between circulating Bcl-2 and other factors in correlation analysis, circulating Bcl-2 can distinguish between CKD and clinically normal age-matched cats *via* PCA.

**Figure 7:**
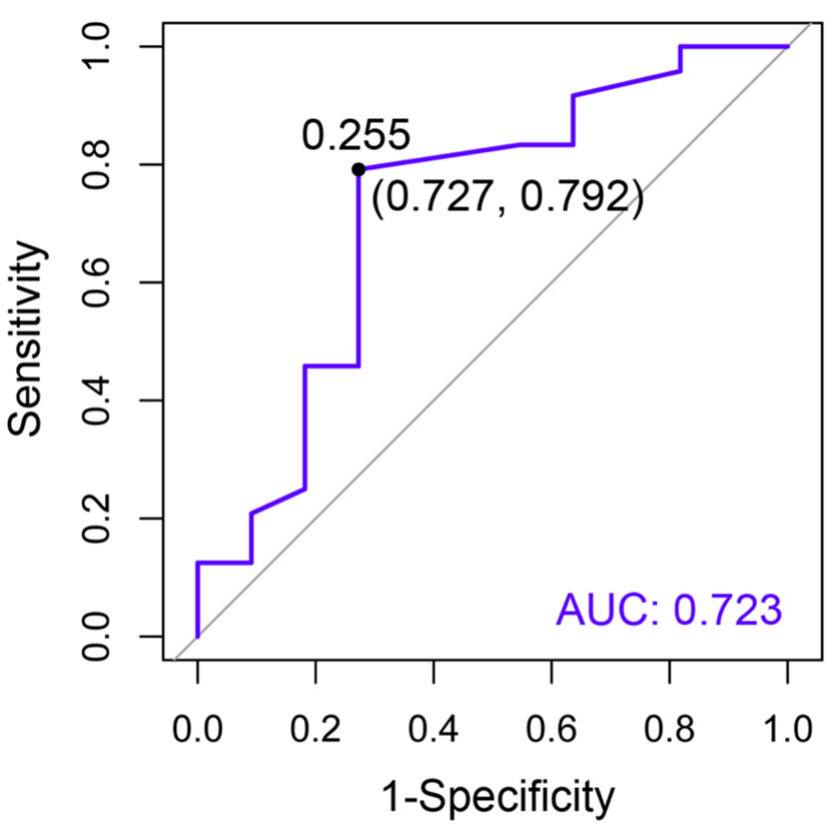
The receiver operating characteristic (ROC) curve of circulating Bcl-2 for the purposes of CKD diagnosis. The specificity (x-axis) is displayed vs. the sensitivity (y-axis). Moreover, AUC is an area under the ROC curve.

The area under the curve (AUC) of circulating Bcl-2 for the detection of CKD was 0.723, indicating an acceptable predictor (Figure 7). The Youden index determined the cutoff threshold for circulating Bcl-2 in cats with CKD and clinically healthy age-matched cats in order to identify those at high risk. When the optimal value of the Youden index was 0.255, this feature was understood to possess optimal performance (sensitivity: 0.727; specificity: 0.792, Figure 7).

**Figure 8:**
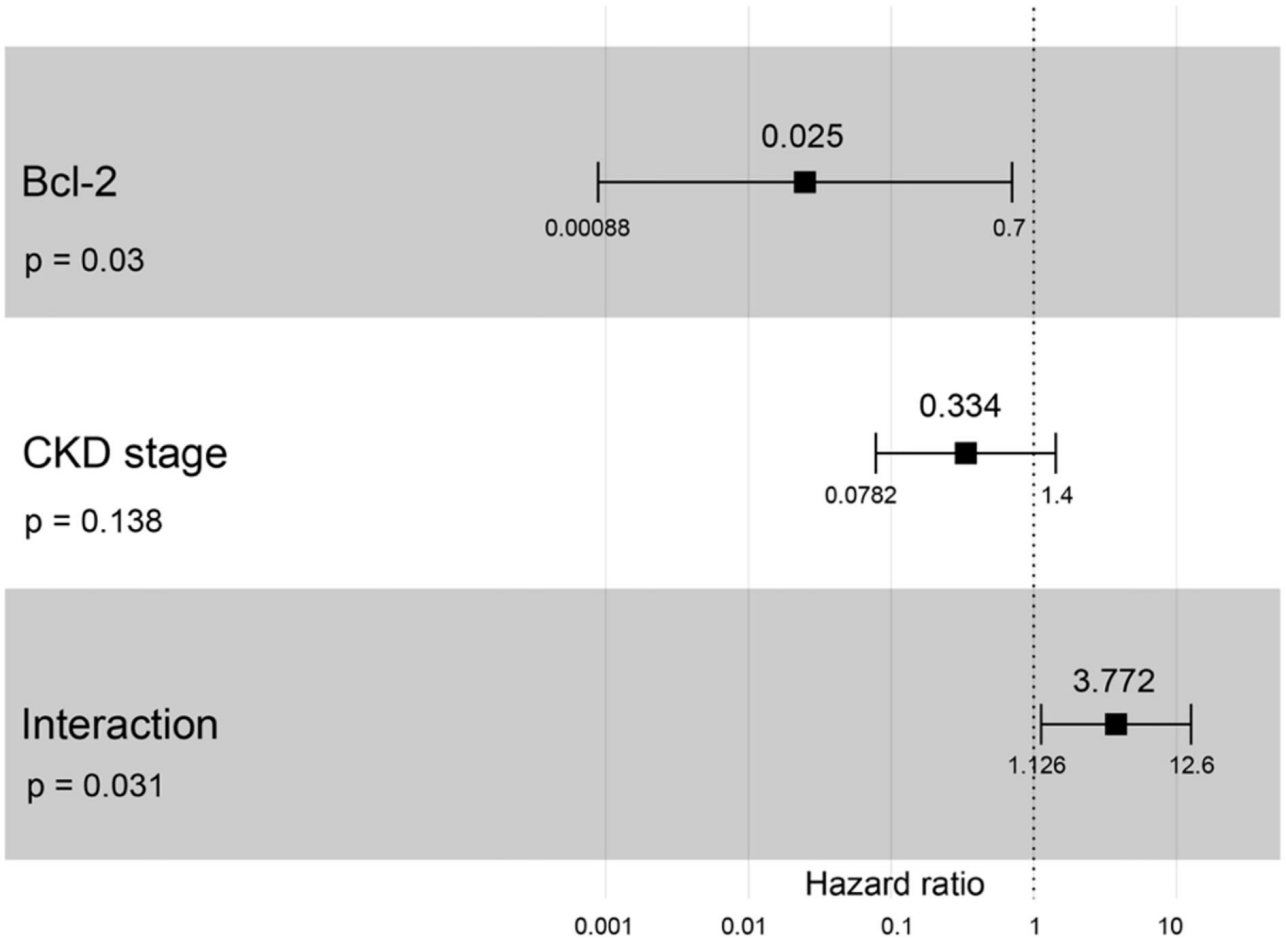
Forest plot output for the interaction of Bcl-2 with CKD stage is covariate with CKD progression based on hazard ratios (HRs) as determined *via* Cox regression analysis. Squares represent hazard ratios. Bars represent 95% confidence intervals.

**Figure 9:**
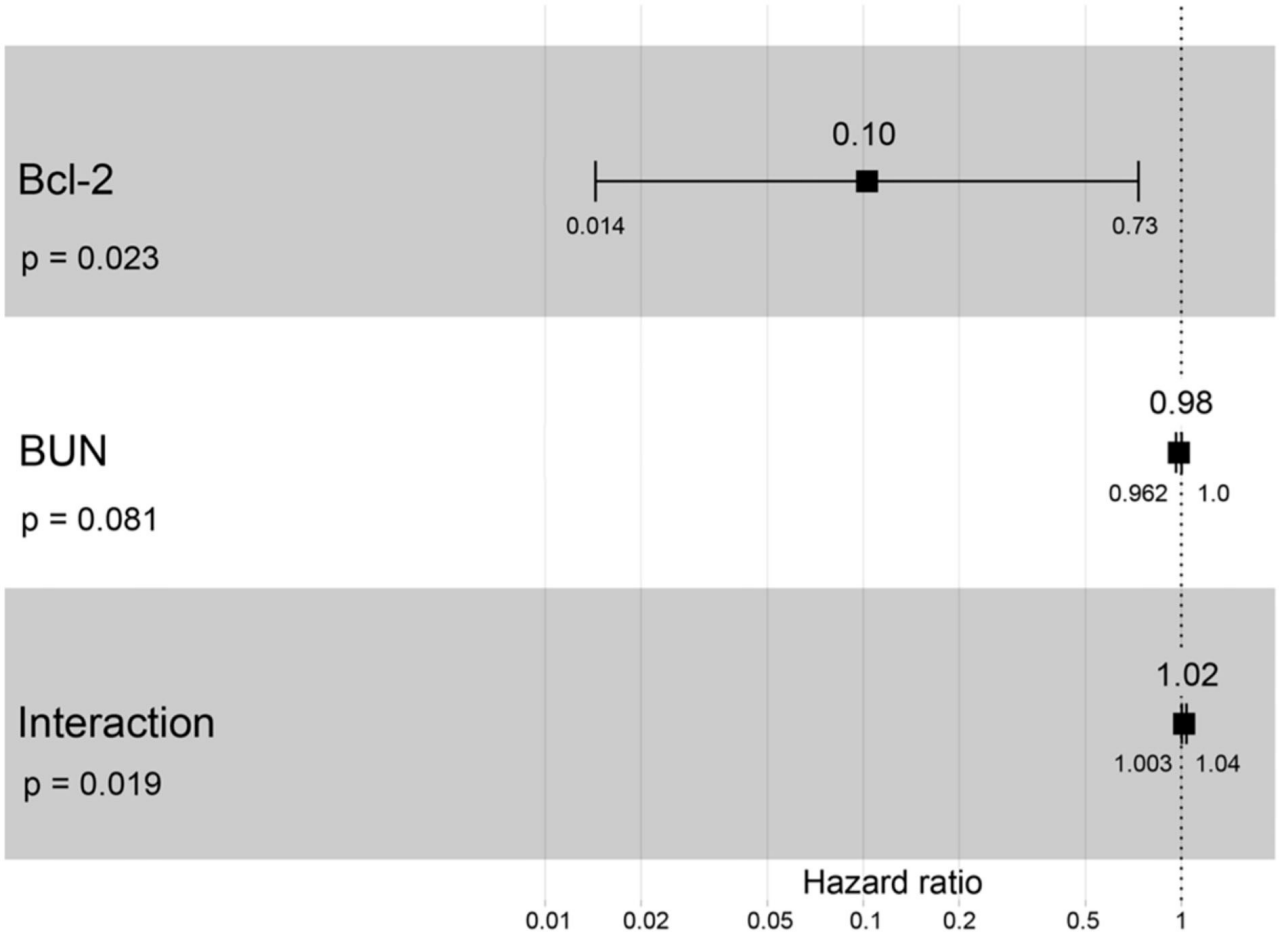
Forest plot output for the interaction of Bcl-2 with BUN that is covariate with the CKD progression based on hazard ratios (HRs) *via* Cox regression analysis. Squares represent hazard ratios. Bars represent 95% confidence intervals.

**Figure 10:**
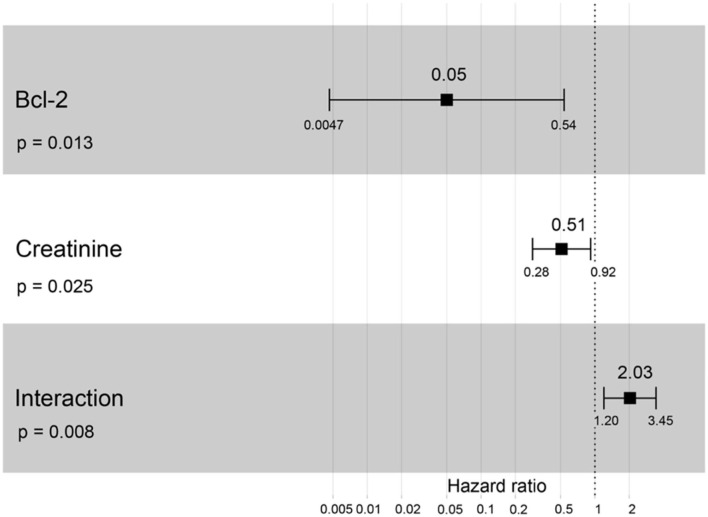
Forest plot output for the interaction of Bcl-2 with creatinine that is covariate with the CKD progression based on hazard ratios (HRs) *via* Cox regression analysis. Squares represent hazard ratios. Bars represent 95% confidence intervals.

**Figure 11:**
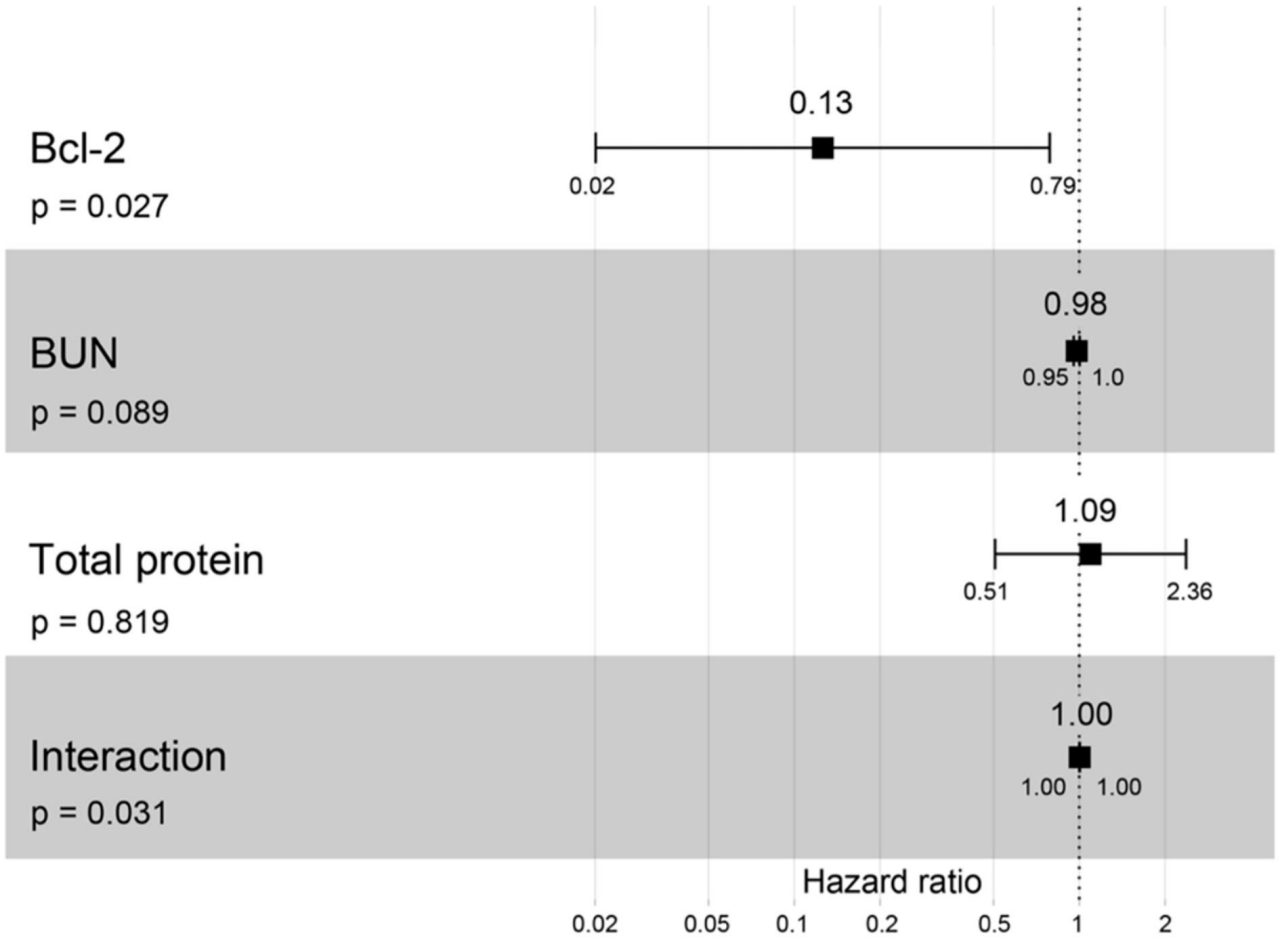
Forest plot output for the interaction of Bcl-2 with two different covariates (BUN and total protein) with CKD progression based on hazard ratios (HRs) *via* Cox regression analysis. Squares represent hazard ratios. Bars represent 95% confidence intervals.

**Figure 12:**
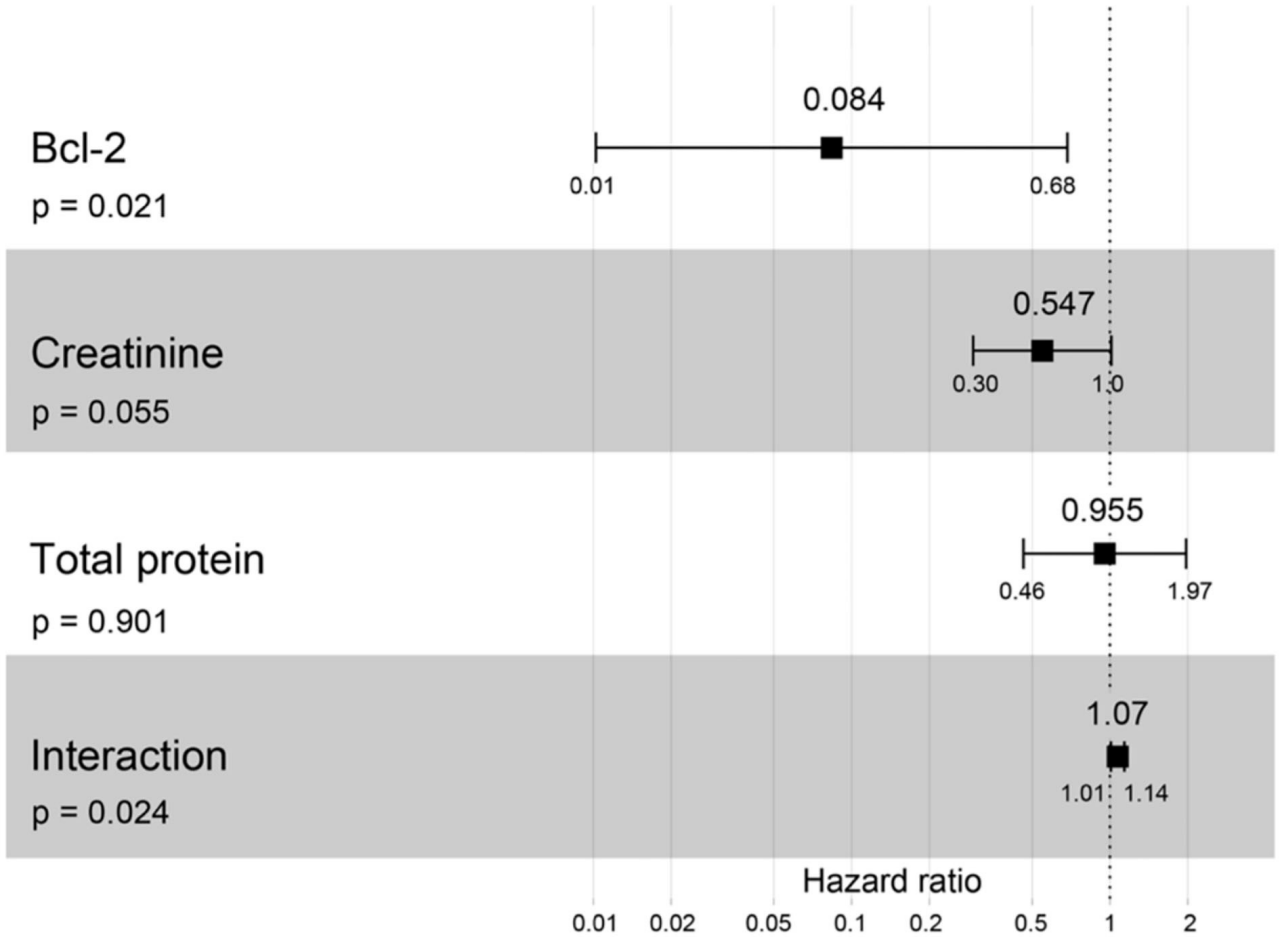
Forest plot output for the interaction of Bcl-2 with two different covariates (creatinine and total protein) with CKD progression based on hazard ratios (HRs) *via* Cox regression analysis. Squares represent hazard ratios. Bars represent 95% confidence intervals.

A hazard ratio greater than one, sex, older age, higher body weight, higher creatinine, higher CKD stage, and higher total protein resulted in an increased risk of developing CKD (Table 2). All variables were included in order to analyze the final models of the interaction between each factor and CKD progression. Figures 8–12 depict the final models of the association between Bcl-2 and other variables for CKD progression. All models indicated that circulating Bcl-2 without interaction with other factors was a risk for CKD progression (Figures 8–12). Moreover, serum creatinine without interaction with other factors in the model was at high risk for CKD progression (Figure 10). The final models of the association between Bcl-2 and CKD stage, as well as between Bcl-2 and serum creatinine had hazardous factors for CKD progression due to the fact these models had a hazard ratio of more than 1 (Figures 8, 10). However, the other final models include Bcl-2 interaction with serum BUN (Figure 9); Bcl-2 interaction with serum BUN and total protein (Figure 11); and Bcl-2 interaction with creatinine and total protein (Figure 12). All of these possessed a hazard ratio equal to 1. As such, these models were at no risk for CKD progression (Figures 9, 11, 12).

**Table 2:** The parameters that are associated with CKD progression (hazard ratio > 1).

**Parameter**	**Beta**	**HR**	**LowerCI**	**UpperCI**	**Wald.test**	***P* value**
BCL2	−1.1	0.33	0.097	1.1	3.3	0.071
Sex	0.11	1.1	0.48	2.6	0.07	0.79
Age	0.057	1.1	0.95	1.2	1.1	0.3
BW	0.062	1.1	0.77	1.5	0.14	0.71
Hct	−0.0061	0.99	0.94	1.1	0.05	0.83
BUN	0.0049	1	1	1	2.9	0.089
Creatinine	0.1	1.1	0.97	1.3	2.3	0.13
Stage	0.49	1.6	1.1	2.5	5.3	0.022
ALT	−0.029	0.97	0.94	1	2.7	0.1
ALP	0.0023	1	0.97	1	0.02	0.89
TP	0.45	1.6	0.83	3	1.9	0.17
Albumin	−0.27	0.76	0.17	3.5	0.12	0.73

ALP, Alkaline phosphatase; ALT, Alanine transaminase; BUN, blood urea nitrogen; BW, Body weight; CI, confidence interval; CKD, Chronic Kidney Disease; Hct, Hematocrit; TP, Total protein.

**Figure 13:**
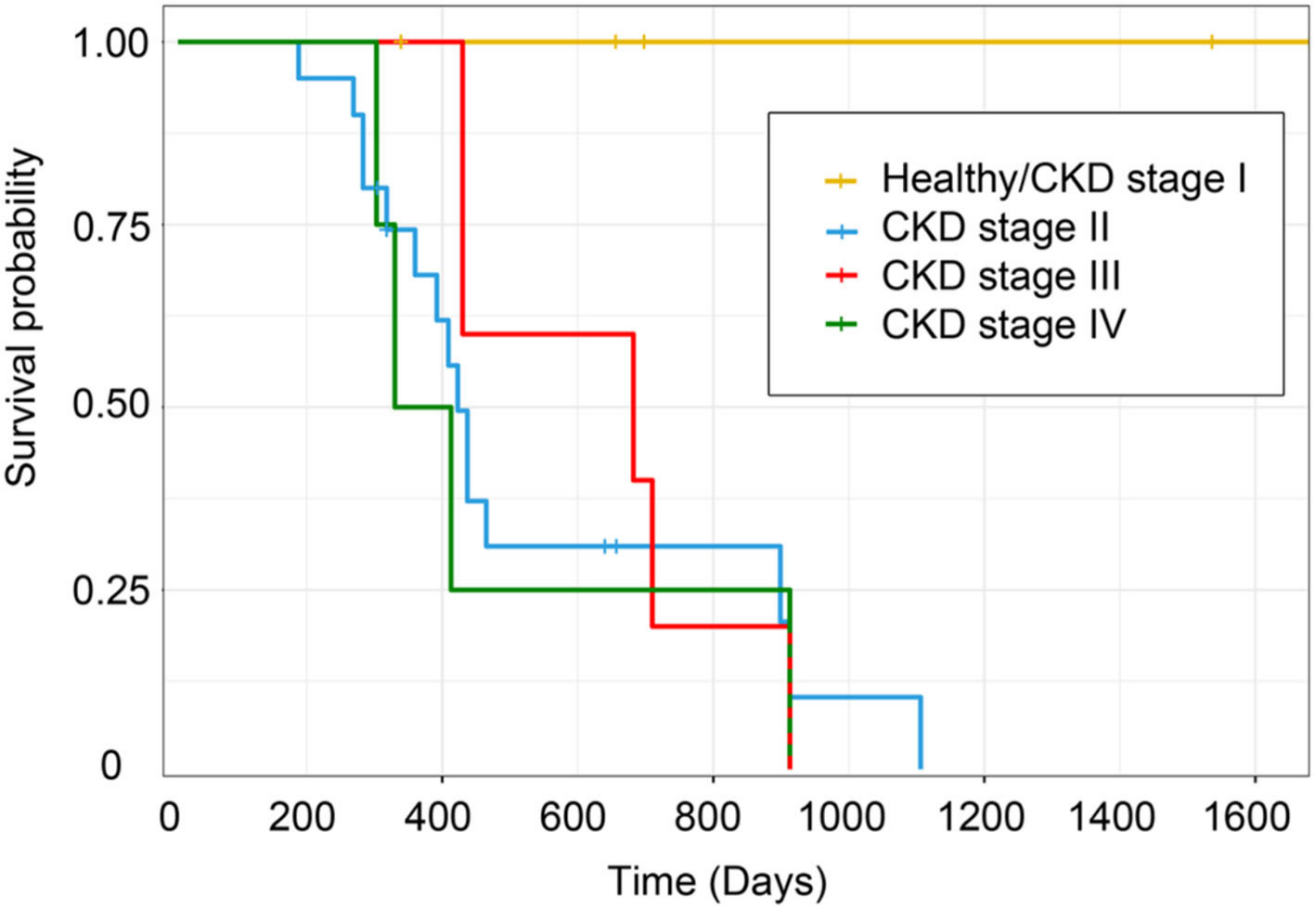
Kaplan–Meier survival estimates by CKD stage. The survival curve of each CKD stage shows the survival time based on the staging of CKD.

Clinically healthy age-matched cats will have the most extended lifespan (< 1,600 days) from the date of diagnosis to the date of analysis. Likewise, cats with CKD stage II will live around 1,100 days, but cats with CKD stages III and IV will live to only about 900 days, *P* = 0.02 (Figure 13). Cats with CKD stage II had a higher survival time than cats with CKD stages III and IV. Moreover, clinically healthy age-matched cats had a significantly higher survival time than cats with CKD stages II–IV.

## Discussion

The present study demonstrated that serum BUN and the creatinine concentration of CKD cats were significantly higher than those found in clinically normal age-matched cats. Other studies have also found that CKD cats increased BUN and creatinine (5, 15, 16). The hematocrit of CKD cats was considerably lower than that of clinically healthy age-matched cats, including the CKD cats that had a reduced blood hematocrit owing to erythropoietin deficiency (17, 18). In this study, the CKD stage correlated with leukocytosis. In addition, increased WBC levels in the Chinese population with normal kidney function can predict future declines in renal function (19). Moreover, the CKD stage possessed a negative correlation with body weight. Piyarungsri et al. (5) also demonstrated that most CKD cats are affected by weight loss and anorexia.

In addition, the CKD stage was negatively correlated with anemia. Erythropoietin deficiency in kidney insufficiency may explain this result (17, 20). In this study, the survival time of cats with the higher stage of CKD was lower than in the early stage. In agreement with the previous research, CKD cats with stage IIb possessed a higher survival time period than cats with stage III and stage IV CKD (21). Increased serum creatinine and BUN, proteinuria, and decreased PCV were all associated with influencing survival time in cats with CKD (22). As such, these results confirmed consolidated knowledge related to CKD.

The present study indicated Bcl-2 was weakly expressed in Bowman's capsule, proximal and distal convoluted tubule, and the collecting duct epithelium cells. The previous studies also reported similar results in other species (8, 9, 23–26). UUO studies demonstrated Bcl-2 expressed in the tubular cells of rats (8) and mice (9). The Bcl-2 was stained in proximal tubules, distal tubules, and interstitial cells in glomerulonephritis patients (23). Bcl-2 expression was strong in mildly dilated tubules and weak in severely dilated tubules of rats with ureteral obstruction (24). Bcl-2 was increased in regard to staining within distal tubules but decreased in the proximal tubules of rats with acute ischemic renal failure (25). Moreover, the Bcl-2 expression had significantly decreased in the renal tissue of diabetic nephropathy patients (26).

The current study investigated whether the circulating Bcl-2 in the CKD cats was significantly lower than those found in clinically normal age-matched cats. In agreement with previous studies, decreased Bcl-2 was also detected in other species with kidney problems (8–10, 27). According to Zhang et al. (8), Bcl-2 immunostaining in the kidney tissues of rats with UUO was less detectable than in the control group. Gene expression levels of Bcl-2 significantly decreased in the renal tissue of monosodium glutamate (MSG)-induced rats compared to the control group (27). Consistent with a recent mouse study, Bcl-2 mRNA was substantially expressed in the UUO3 group but decreased in the UUO7 and UUO14 groups (9). Additionally, a prior investigation on human patients found that Bcl-2 levels were lower in patients with end-stage renal disease patients than in individuals with chronic kidney disease (10).

The Bcl-2 protein was one of the self-protection mechanisms induced by damaged cells (8). Another study reported that Bcl-2 preserve the mitochondrial membrane and binds to Bax in order to inhibit apoptosis in leukemias (28). In addition, wounded cells express both anti- and pro-apoptotic proteins, but injured cells will survive if an increase in Bcl-2 is achieved (9). The results of the present study substantiated Bcl2's anti-apoptotic involvement in chronic renal disease in cats. The involvement could be clarified by previous studies (27, 29, 30). The down-regulation of Bcl-2 plays a role in renal cell death (27). Decreased Bcl-2 may enhance ischemia/reperfusion-induced oxidant stress, as well as tubular apoptosis (29). Renal tubular apoptosis, by the decreasing in Bcl-2, was associated with tubular atrophy and chronic renal fibrosis (30). Thus, Bcl-2 may be related to kidney disease through apoptosis.

Although the current study indicated that circulating Bcl-2 did not relate to BUN and creatinine, lower circulating Bcl-2 with higher BUN, creatinine, and CKD severity was associated with CKD progression. The present study is a limited cohort size that may affect no correlation between circulating Bcl-2 and other parameters. However, more sample sizes in the previous studies reported the same trend as the present study. Forty patients with renal diseases possessed lower Bcl-2 gene expression in their blood samples than in 20 healthy individuals (10). As previously reported, there was no correlation between Bcl-2 positive cells and BUN or creatinine levels in glomerulonephritis patients (31). However, Bcl-2 was related to hypertension and glomerular hypertrophy, which could develop CKD (32). Further study is needed in order to include more clinically healthy cats and CKD cats.

Several studies also reported the AUC of Bcl-2 in other chronic diseases (33–35). Bcl-2 was a significant predictor in epithelial ovarian cancer patients (0.838) (33) and breast cancer patients (AUC = 0.841) (34). Moreover, the Bcl-2/Bax ratio may be a differentiator for human osteoarthritis (AUC = 0.673) (35). This study indicated that the AUC of Bcl-2 for predicting cats with CKD was 0.723, which is a reasonable discrimination ability (36). Thus, it could be evaluated that Bcl-2 has a prediction ability for CKD in cats.

## Conclusions

In conclusion, Bcl-2 levels in CKD cats were considerably lower than those in clinically normal age-matched cats. The AUC analysis of Bcl-2 demonstrated that it adequately predicted CKD in cats. Moreover, in cats, decreased Bcl-2 expression was associated with increased BUN, creatinine, and CKD severity. Unfortunately, the immunostaining intensity of Bcl-2 was weak in the kidney tissues of cats with CKD. This study revealed that Bcl-2 could help with distinguishing between cats with chronic kidney disease and healthy cats. However, the present study is a small sample size, is not fully validated, and has no longitudinal follow-up study. Further study is required in order to include a more substantial sample size, to investigate the validation of these results, to follow up the longitudinal results, to perform on Bcl-2 immunostaining in kidney tissues from cats that died from other diseases, and to better determine the relationship between Bcl-2 levels in kidney tissue and Bcl-2 levels in the blood of live cats.

## Data availability statement

The original contributions presented in the study are included in the article/Supplementary material, further inquiries can be directed to the corresponding author.

## Ethics statement

The animal study was reviewed and approved by the Institutional Animal Care and Use Committee, Faculty of Veterinary Medicine, Chiang Mai University, Chiang Mai, Thailand (reference No. S25/2563, date of approval: 17 July 2020). Written informed consent was obtained from the owners for the participation of their animals in this study.

## Author contributions

KPi and PC designed the study, were involved in the supervision, and edited the manuscript. KPi and PPi recruited cases and organized the sample collection. PC and PPa performed the statistical analysis. KPr provided helpful suggestions and performed the immunohistochemistry. PPi, KPi, and PC performed the assays. PPi wrote the first draft of the manuscript. KPi was involved in funding acquisition. All authors contributed to the article and approved the manuscript.
