# Supplementary material for: Evaluation of Bcl-2 as a marker for chronic kidney disease prediction in cats
Source: Front Vet Sci. 2023 Jan 9;9:1043848. doi: 10.3389/fvets.2022.1043848 (PMC9870326; doi:10.3389/fvets.2022.1043848)
Supplement: Supplementary file 1 [file Data_Sheet_1.docx]

Supplementary Material

# Supplementary Figures


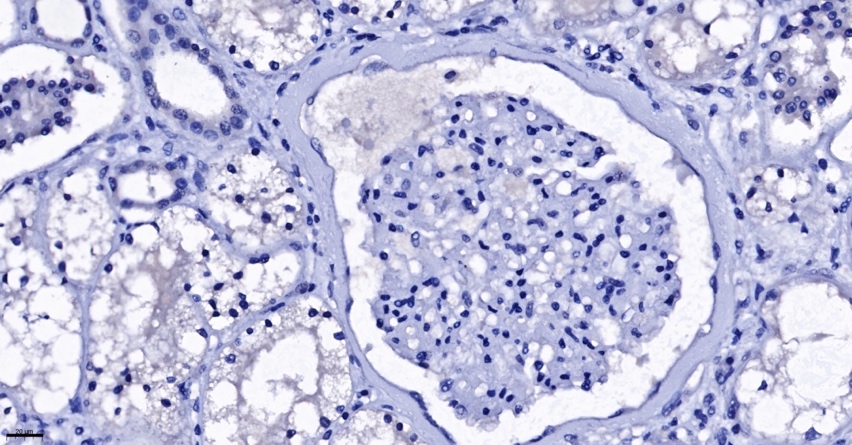


**Supplemental Fig. 1.** The immunohistochemistry staining of Bcl-2 protein in the negative control. Original magnification 400×. Scale bar = 20 µm. Negative control was performed by replacing the primary antibody with the normal mouse serum (1:200).
